# Evaluation of a Child and Adolescent Mental Health Service Using a Multidisciplinary Team Approach for New Assessments of ADHD

**DOI:** 10.1192/bjo.2025.10506

**Published:** 2025-06-20

**Authors:** Emma Mittelman, Abigail Swerdlow, Mark Berelowitz

**Affiliations:** 1 North London NHS Foundation Trust, London, United Kingdom; 2 Royal Free Hospital, London, United Kingdom

## Abstract

**Aims:** To reduce the waiting list, senior Psychologists and Psychotherapists started undertaking ADHD assessment as well as Psychiatrists in a CAMHS Outpatient Clinic. The aim of this Service Evaluation was to see if there was any difference regarding who performed the initial assessment and the management offered.

**Methods:** New assessment letters were reviewed from February–November 2024.

Data was collected including demographics, type of clinician, diagnosis and management.

Categorical data was assessed for statistical significance using Chi-square tests and numerical data using ANOVA.

The data was presented to the MDT to think about clinical significance.

**Results:** 103 patients were assessed with an average age of 11.7. Fifty-four were seen by a Psychiatrist, 39 were seen by a Psychotherapist and 10 were seen by a Psychologist. 25 of these patients required a follow-up with a Psychiatrist.

28 of the patients had a previous diagnosis of ADHD and therefore were required to be seen by a Psychiatrist. Of these patients, 26 retained their diagnosis at the point of initial assessment.

Of the children that had not been previously diagnosed with ADHD, 72% were given a new diagnosis of ADHD at initial assessment. After accounting for previously diagnosed patients, there was no statistical significance in number diagnosed by the different types of clinicians. There was no statistically significant difference between the management options offered and the type of clinician assessing.

**Conclusion:** Since this change, the service was able to nearly double the number of young people seen. This is a vital step as the number of referrals for the service has also increased over this time.

It was felt that there was a consistent approach across the service as there was found to be no statistically significant difference in either diagnosis given or management options offered by clinician types, after accounting for prior diagnosis. This allows some confidence that patients get the same, unbiased approach, regardless of clinician type.

Psychiatrists had a follow-up appointment with about half of the patients assessed by other clinicians. The follow-up takes less clinical time compared with the new assessment, however, this must be considered in the planning of a service.

Once a child has been diagnosed it is crucial that they are able to be offered the necessary services (Family Therapy, Psychology, Psychotherapy, Medication reviews) in a timely manner. Services must ensure a good balance between new assessments and looking after those post diagnosis.

